# Impact of KCl-divalent salt synergistic substitution on protease activity and volatile flavor profile in sodium-reduced sour meat

**DOI:** 10.1016/j.fochx.2026.104294

**Published:** 2026-08-08

**Authors:** Ying Chen, Ning Liu, Yuqian Guan, Shenghui Bi, Lihua Shang, Chaoyue Yang, Cinan Li, Yuanyuan Liu, Binpeng Xu, Qiujin Zhu, Ying Zhou

**Affiliations:** a Guizhou Key Laboratory of New Quality Processing and Storage of Ecological Specialty Food, School of Liquor and Food Engineering, Guizhou University, Guiyang 550025, China; b Key Laboratory Mountain Plateau Animals Genetics and Breeding, Ministry of Education, Guiyang 550025, China; c Postdoctoral Research Station, Anhui Chem-Bright Bioengineering Co., Ltd, Huaibei 235000, China

**Keywords:** Proteases, Key taste-active amino acids, Volatile flavor compounds, Fermented meat

## Abstract

Sour meat is a traditional Chinese fermented product valued for its unique flavor, which is achieved through high-salt processing. Nevertheless, the consequent excessive sodium intake increases the health risks. This study developed a sodium-reduced sour meat strategy by replacing 40% of sodium chloride (NaCl) with potassium chloride (KCl) in combination with divalent salts, namely calcium chloride (CaCl_2_) or magnesium chloride (MgCl_2_). The substitution differentially regulated endogenous proteases: KCl-MgCl_2_ enhanced cathepsin B/L and dipeptidyl peptidase IV activities, increasing volatile compounds; KCl-CaCl_2_ elevated dipeptidyl peptidase I and alanyl aminopeptidase, promoting TCA-soluble peptides and amino acids. PLS-DA identified key flavor compounds (VIP >1), and correlations linked protease activities to flavor formation. The strategy enables targeted enzymatic regulation for producing healthier sour meat with improved flavor.

## Highlights

• A novel low-sodium salt substitution strategy was developed and validated.
• Non-sodium salts specifically regulated protease activity and proteolysis.
• Key taste-active amino acids including Glu, Leu and Lys were characterized.
• Critical volatiles with VIP > 1 like 4-isopropylbenzaldehyde were identified.
• Correlations among proteases, amino acids and key volatiles were revealed.

## Graphical abstract

**Figure:**
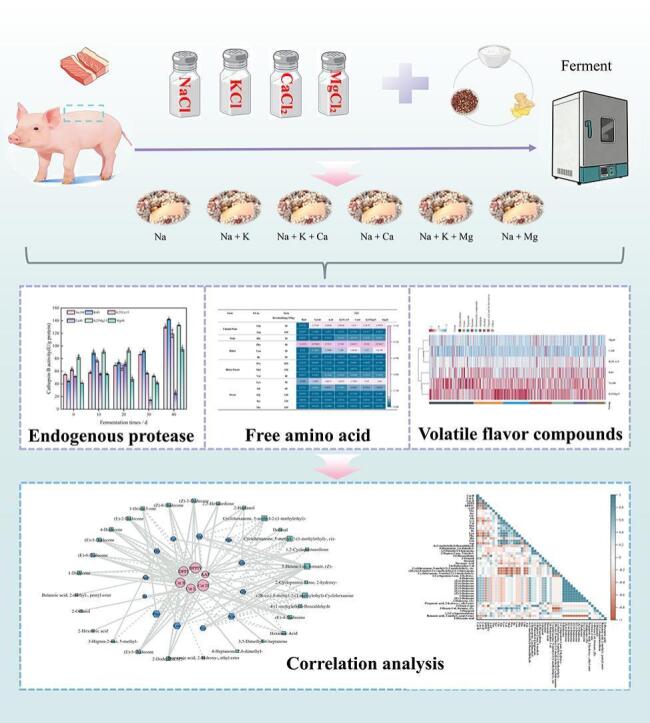

## 1 Introduction

Sour meat, a traditional fermented meat product distinguished by its distinctive flavor profile and nutritional quality, is widely consumed among ethnic communities in the Hunan and Guizhou provinces of China. Traditionally, sour meat is prepared by mixing fresh meat with salt, rice, and spices, followed by anaerobic fermentation under sealed conditions (Lv et al., 2023). During this process, proteins, lipids, and carbohydrates are broken down into amino acids and free fatty acids, thereby enhancing digestibility and nutrient absorption efficiency (Zeng et al., 2021; Zhang, Hu, et al., 2020). However, to maintain sensory quality and extend shelf life, traditional sour meat typically contains 4%–8% (w/w) sodium chloride (NaCl) (Meng et al., 2025). NaCl inhibits undesirable microorganisms by reducing water activity (Aw), thereby ensuring the safety of the sour meat fermentation system. Additionally, NaCl regulates endogenous enzyme activities, modulates proteolytic reactions, and systematically generates flavor precursors such as free amino acids (FAAs), playing a crucial role in the formation of the characteristic flavor of sour meat. However, substantial research has confirmed that high sodium intake is closely associated with an increased risk of hypertension, cardiovascular diseases, and several cancers (Campbell et al., 2007; Hu et al., 2011). Consequently, the World Health Organization recommends a daily sodium intake not exceeding 2 g (He & MacGregor, 2018). According to the survey data from the Chinese Center for Disease Control and Prevention, the average daily sodium intake among Chinese adults reaches 4.18 g, with 89.4% exceeding the recommended limit. As a traditional food in Southwest China, sour meat further aggravates the regional sodium intake burden. Under the national “Three Reductions and Three Health” initiative and the “Salt Reduction Guidelines for the Food Industry,” there is an urgent practical need to develop sodium-reduced sour meat strategies that balance both health and flavor.

Currently, common salt reduction strategies in fermented meat products include the use of salt substitutes, flavor enhancers, and novel non-thermal processing technologies. Among these strategies, partial replacement of NaCl with non‑sodium salts such as potassium chloride (KCl), calcium chloride (CaCl_2_), and magnesium chloride (MgCl_2_) represents the most established technological approach for sodium reduction. KCl has been extensively studied due to its salty taste profile and the potential antihypertensive effects of potassium. However, when the substitution level exceeds 40%, this can adversely affect the texture and flavor of fermented meat (dos Santos et al., 2015). Introducing divalent salts such as MgCl_2_ and CaCl_2_ on the basis of KCl substitution not only helps reduce sodium content but also supplements essential minerals like magnesium and calcium, thereby helping prevent diseases such as osteoporosis (Cittadini et al., 2019).

Endogenous protease, mainly including cathepsins, dipeptidyl peptidases (DPPs) and aminopeptidases (Li et al., 2024; Zhang et al., 2025), are recognized as key participants in proteolytic reactions within fermented meat products (Verplaetse, 1994; Wang et al., 2024). During fermentation, cathepsins act upstream by degrading myofibrillar proteins into polypeptides; DPPs function in the midstream by further cleaving the polypeptides into small peptides; and aminopeptidases work downstream by hydrolyzing the small peptides into FAAs (Hu et al., 2023). These small peptides and amino acids are essential for the taste of sour meat and function as key precursors for volatile organic compounds (VOCs) formation (Guo et al., 2021). Research indicates that approximately 8.3% - 18.1% of VOCs in fermented meat originate from proteolytic reactions. FAAs generated through proteolysis can be further transformed into volatile flavor components via pathways such as the Maillard reaction and Strecker degradation (Picon & Nuñez, 2022). Notably, moderate protein degradation enhances saltiness perception and improves the product's dietary value, whereas excessive degradation may lead to quality deterioration, off-flavors, and even the formation of undesirable substances like biogenic amines (Dong et al., 2024). These findings collectively indicate that endogenous proteases play a critical role in the proteolysis and flavor formation of sour meat.

Nevertheless, salt content and composition are key parameters regulating enzymatic activity, significantly influencing the functionality of endogenous enzymes in fermented meat products (Li et al., 2025; Zhang, Yang, et al., 2020). Aspholm et al. (2024) demonstrated that diverse divalent metal ions exert bidirectional regulation over the activity of mitochondrial serine protease HtrA2. Specifically, Mg^2+^ and Ca^2+^ activate the enzyme, while Zn^2+^ and Cu^2+^ suppress catalytic capacity, enabling precise modulation of enzymatic activity. This demonstrates that the regulatory effects of divalent metal ions on proteases vary with ion species. Similarly, salt ions may modulate the activities of endogenous proteases in meat during fermentation. Zhang, Yang, et al. (2020) reported that partial replacement of NaCl with CaCl_2_ and MgCl_2_ in dry-cured pork butt enhanced cathepsin activity to a certain degree, further verifying this point. Existing research on fermented meat have demonstrated that integrating biochemical, microbial, and metabolomic analyses provides a comprehensive understanding of flavor formation during fermentation. These approaches linked microbial succession and metabolite dynamics to characteristic sensory development, highlighting the complexity of flavor generation (Wang, Li, et al., 2022). Nevertheless, most studies on sodium reduction in sour meat have emphasized microbiomic and metabolomic perspectives, while neglecting endogenous enzyme activities and their relationship to flavor formation.

Based on the above analysis, we hypothesize that different salt ions (K^+^, Ca^2+^, and Mg^2+^) may differentially regulate the activities of cathepsins, DPPs, and aminopeptidases, thereby altering the conversion efficiency of polypeptides into small peptides and FAAs within the proteolytic cascade. This, in turn, may affect the composition and content of FAAs in sour meat, ultimately leading to differences in the formation of volatile flavor compounds. To verify the above hypothesis, this study employed multiple analytical techniques including multiple analytical approaches, including enzymatic activity assays, proteolysis index (PI), FAAs profiling, and volatile compound analysis, to systematically explore how partial NaCl substitution (40%) with KCl combined with CaCl_2_ or MgCl_2_ alters endogenous protease activity and further modulates volatile flavor generation. Fermentation was performed at 15 °C and 80% RH, consistent with typical processing parameters for traditional Guizhou fermented sour meat. By clarifying the divergent regulatory pathways of different salt formulations on flavor formation from the perspective of endogenous protease dynamics, this study provides theoretical support for the quality optimization and industrial manufacture of sodium-reduced fermented meat products.

## 2 Materials and methods

### 2.1 Sour meat processing

Sour meat was produced using pork loin sourced from Guizhou Tainong Xingwang Food Co., Ltd. (Guiyang, China). The animals were raised for over one year on a diet consisting mainly of corn, sorghum, and soybeans, reaching a live weight of around 125 kg at the time of slaughter. The pork loin was sliced into pieces measuring (8 ± 1) cm × (6 ± 1) cm × (1 ± 0.2) cm. Subsequently, salt mixtures with different formulations were added at 4% (w/w) of the meat weight, following the specific formulations in Table 1.

**Table 1:** Sour meat curing formula.

Experimental group	NaCl	KCl	CaCl_2_	MgCl_2_
Na100	100%			
K40	60%	40%		
K25Ca15	60%	25%	15%	
Ca40	60%		40%	
K25Mg15	60%	25%		15%
Mg40	60%			40%

The mixture was tumbled for 1 h using a meat tumbler, followed by static marination for 1 h. Afterwards, 2% (w/w) pre-roasted aromatic Sichuan pepper and 40% (w/w) pre-roasted glutinous rice (until golden yellow) were added based on the meat weight and thoroughly mixed. The mixture was then vacuum-packed and fermented in a constant temperature and humidity incubator (temperature: 15 °C, relative humidity: 80%). Samples were collected on days 0, 10, 20, 30, and 40 of fermentation. After sampling, the glutinous rice coating, excess fat, and connective tissue were removed, and the samples were stored at −80 °C.

### 2.2 Determination of physical and chemical properties

#### 2.2.1 pH and total salt content

A 1 g minced meat was diluted tenfold with ultrapure water and homogenized at 4500 rpm for 1 min. The pH value and total salt content of the sour meat sample were then measured. The salinity meter operates based on the conductivity method, which converts the total electrical conductivity of the solution into salt content. The result reflects the sum of the contents of various salts (NaCl, KCl, CaCl_2_, and MgCl_2_) in the sample.

#### 2.2.2 Aw

A 5 g sample of minced sour meat was evenly spread in a measurement dish, and the Aw of the sample was subsequently determined using a water activity meter.

#### 2.2.3 Moisture content

A 3 g meat sample was chopped and evenly distributed in a measurement dish. The moisture content was assessed with a dedicated meat moisture analyzer (SFY-30, Guanya, Shenzhen, China).

### 2.3 Endogenous protease activity

#### 2.3.1 Extraction of crude protease

Cathepsin crude enzyme extraction was performed according to Zhang, Bi, et al. (2024) with minor modifications. A 5 g sample (fat and connective tissue removed) was homogenized with four volumes (w/v) of extraction buffer (50 mmol/L Tris-HCl, pH 7.5; 10 mmol/L β-mercaptoethanol; 1 mmol/L EDTA) at 7000 rpm for 30 s. The homogenate was stirred at 4 °C for 1 h and centrifuged at 10,000*g* for 25 min at 4 °C. The supernatant was collected and filtered. The same protocol was used to extract dipeptidyl peptidase I (DPP I), dipeptidyl peptidase IV (DPP IV), and alanyl aminopeptidase (AAP), except the buffer was replaced with 50 mmol/L phosphate (pH 7.5) containing 5 mmol/L EGTA (Li et al., 2024).

Cathepsin D was extracted following the method of Tian et al. (2013) with slight adaptations. A 5 g meat sample was homogenized with two volumes (*w*/*v*) of pre-cooled extraction buffer (50 mmol/L sodium acetate, 7 mmol/L cysteine, 0.9 mmol/L PMSF, pH 5.0), stirred at 4 °C for 2 h, and centrifuged at 12,000*g* for 30 min at 4 °C. The supernatant was collected for activity assay.

#### 2.3.2 Determination of endogenous protease activities

Cathepsin B and L activities were measured following a previously described method (Zhang, Bi, et al., 2024). For cathepsin B, the 2.5 mL substrate solution contained 50 mM sodium phosphate buffer (pH 6.0), 0.3125 mM *Z*-Arg-Arg-AMC, 4 mM EDTA, 2 mM DTT, and 3.4 mL/L Brij-35. For combined cathepsin B and L activity, Z-Phe-Arg-AMC was used instead. The reaction mixture was incubated with 0.5 mL of the enzyme extract at 37 °C for 20 min and terminated by adding 0.6 mL of absolute ethanol. One unit (U) was defined as the amount of enzyme releasing 1 nmol of AMC per minute at 37 °C. Protein concentration in crude enzyme extracts was quantified using a BCA kit. DPP I, DPP IV, and AAP activities were assayed according to Li et al. (2024) with minor modifications, using Gly-Arg-AMC, Gly-Pro-AMC, and Ala-AMC as specific substrates, respectively.

Cathepsin D activity was assayed in a 1000 μL reaction mixture containing 250 μL hemoglobin (0.02 g/mL), 250 μL acetate buffer (0.2 mmol/L, pH 3.6), and 500 μL enzyme extract. After incubation at 37 °C for 30 min, the reaction was stopped with 125 μL of 10% trichloroacetic acid (TCA), followed by centrifugation at 8000*g* for 10 min. The absorbance of the supernatant was measured at 280 nm. One unit (U) was defined as the amount of enzyme causing an absorbance increase of 0.01 per minute per gram of protein.

### 2.4 Protein hydrolysis determination

#### 2.4.1 Determination of PI

The PI was quantified according to the fluorometric method described by Harkouss et al. (2012). A 0.7 g meat sample was homogenized with 7 mL of cold distilled water using a Polytron PTMR 2100 homogenizer (30s, 12,000 rpm). Then, 150 μL of the homogenate was mixed with 600 μL of 12.5% TCA to precipitate proteins, agitated for 15 min at 4 °C, and centrifuged at 2000×*g* for 10 min. Peptide and amino acid concentrations in the supernatant were determined following Harkouss et al. (2012). Briefly, 300 μL of supernatant was neutralized with 300 μL of 2 M NaOH (pH 10) to reach pH 9.0. Then, 180 μL of fluorescamine solution (0.6 mg/mL in acetone) was added, and the mixture was incubated in the dark at 25 °C for 1 h. Fluorescence was measured at excitation/emission wavelengths of 375/475 nm, with background fluorescence subtracted. A glycine standard curve (5–50 mM) was used for quantification. Total protein content in the homogenate was determined by BCA assay prior to TCA precipitation. The PI was expressed as the percentage ratio of α-amino groups to total protein.

#### 2.4.2 Trichloroacetic acid-soluble peptides (TCA-soluble peptides) assay

A 2 g sample of minced meat were blended with 18 mL of 5% TCA in an ice bath. Following incubation at 4 °C for 1 h and centrifugation (6000×*g*, 15 min), the supernatant was collected. TCA-soluble peptide concentration was quantified via the Lowry method and reported as μmol Tyr/g (Liu et al., 2025).

#### 2.4.3 FAAs analysis

The determination of FAAs content using the modified method of Zhong et al. (2025). The filtrate was obtained by homogenizing 1 g of sample in 50 mL of 0.01 M HCl for 30 min. Subsequently, 2 mL of filtrate was mixed with an equal volume of 8% sulfosalicylic acid, centrifuged at 10,000×*g* for 10 min, and filtered. FAAs were analyzed using an amino acid analyzer (HITACHI L-8900, Tokyo, Japan).

### 2.5 Analysis of VOCs

#### 2.5.1 Headspace solid-phase microextraction (HS-SPME) analysis

Frozen sour meat samples were ground in liquid nitrogen. 0.2 g powder and 0.2 g NaCl (to inhibit enzymes) were placed into a 20 mL headspace vial (Agilent) and sealed. After equilibration at 60 °C for 5 min, volatiles were extracted using an SPME Arrow fiber (120 μm DVB/CWR/PDMS, Agilent) at 60 °C for 15 min.

#### 2.5.2 Gas chromatography-mass spectrometry (GC–MS) conditions

Desorption: 250 °C, 5 min. GC (Agilent 8890) with DB-5MS column (30 m × 0.25 mm × 0.25 μm), He carrier 1.2 mL/min. Oven: 40 °C held for 3.5 min, then increased at 10 °C/min to 100 °C, then at 7 °C/min to 180 °C, then at 25 °C/min to 280 °C held for 5 min. MS (7000D): EI 70 eV; quadrupole 150 °C, ion source 230 °C, transfer line 280 °C; SIM mode.

VOCs were identified by mass spectral matching (MWGC) and linear retention indices. For each compound, one quantitative and two or three qualitative ions were sequentially detected in time segments by elution order. Identification was confirmed by comparing retention times with standards and verifying that the background-subtracted spectrum contained all characteristic ions. For accurate quantification, specific ions were used for integration. Using 3-hexanone (10 μL, 50 μg/mL) as internal standard, relative contents were calculated as:

$$\mathrm{Cj}=\left(\mathrm{Aj}\times \mathrm{mi}\right)/\left(\mathrm{Ai}\times \mathrm{ms}\right)$$

Cj is the mass concentration (μg/kg) of the compound; Aj and Ai are the peak areas of the compound and internal standard; mi and ms are the masses of internal standard (μg) and sample powder (kg).

### 2.6 Statistical analysis

The data are expressed as mean ± standard error (SE) from at least three independent experiments. Significant differences were evaluated using one-way analysis of variance (ANOVA), followed by Duncan's multiple range test at *p* < 0.05. All statistical analyses were performed with SPSS software, while figures were generated using Origin and SIMCA 14.1 software (for partial least squares discriminant analysis, PLS - DA).

## 3 Results and discussion

### 3.1 Changes in physicochemical properties during fermentation

**Fig. 1:**
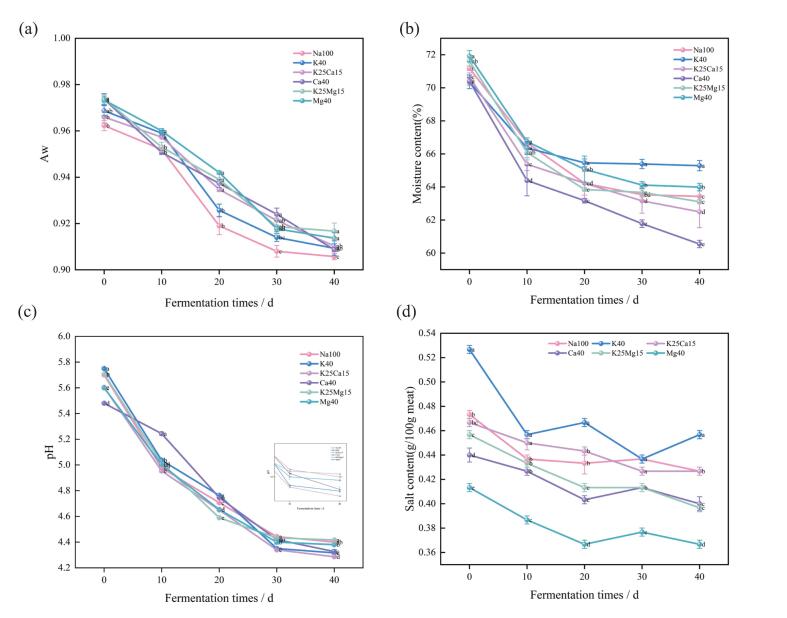
Changes of physiochemical properties of sour meat during fermentation. (a) Aw; (b) Moisture content; (c) pH; (d) Salt content. Note: Different lowercase letters indicate significant differences at *p* < 0.05.

As seen in Fig. 1a, the Aw gradually decreased with prolonged fermentation time. By day 40, the Aw in all experimental groups had dropped to ≤0.92. This value is near or below the minimum Aw threshold (0.90–0.95) required for the growth of most spoilage microorganisms, indicating that the product had reached the endpoint of fermentation. At day 40, the Aw values in the salt-replacement groups were significantly higher than that in the full-salt (NaCl) control group (*p* < 0.05). Notably, the K25Mg15 substitution group exhibited a significantly higher Aw compared with the other experimental groups (*p* < 0.05). These results indicate that partially replacing NaCl in sour meat with non‑sodium salts such as KCl, CaCl_2_, and MgCl_2_ can increase the product's Aw.

As observed in Fig. 1b, the moisture content also exhibited a gradually decreasing trend. A sharp decline occurred between day 0 and day 10, followed by a more gradual decrease from day 10 to day 40. Furthermore, during the period from day 20 to day 40, the 40% KCl substitution group maintained the highest moisture content. This could be due to the quicker penetration of the salt mixtures containing KCl that would hinder the exit of water from the inside of the meat (Aliño et al., 2009; Wu et al., 2014). Conversely, the 40% CaCl_2_ substitution group showed the lowest moisture content. This phenomenon can be explained by the dehydrating property of CaCl_2_, as calcium ions enhance mass transfer, resulting in a higher dehydration rate (Vidal et al., 2020). This suggests that replacing NaCl with KCl can mitigate the loss of moisture in sour meat, while using CaCl_2_ exacerbates it. In addition, the moisture content of the K25Mg15 and K25Ca15 substitution groups was slightly lower than that of the control group. This demonstrates that co-substituting NaCl with KCl and CaCl_2_ or MgCl_2_ can slightly reduce the product's moisture content.

As observed in Fig. 1c, the pH value gradually decreased with the extension of fermentation time. The pH reduction might result from microbial utilization of carbohydrates, generating acidic metabolites such as lactic acid during metabolic activity (Sun et al., 2016). The dynamic changes in pH can indirectly reflect the accumulation and metabolism of organic acids (mainly lactic acid). During the first 10 days of fermentation, the rapid decrease in pH may provide a suitable acidic environment for cathepsins B/L, thereby facilitating the initiation of proteolysis in sour meat. On day 40 of fermentation, no significant difference was found in the pH values of the K25Mg15 and Mg40 substitution groups, when compared with the control group. However, the pH value of sour meat in K25Ca15 and Ca40 substitution groups decreased significantly, which indicated that calcium ions could significantly affect the pH value of sour meat. This outcome aligns with the observations reported by Zhang, Yang, et al. (2020), who observed a similar trend in the pH of dry-cured pork butts when sodium was partially replaced by different metal ions.

As seen in Fig. 1d, the salt content in the Ca40 and Mg40 substitution groups was significantly lower than that in the K40 and Na100 groups (*p* < 0.05). This difference may be attributed to the slower permeation and diffusion rates of divalent metal ions compared with monovalent ions. This may be attributed to the higher charge densities of Ca^2+^ and Mg^2+^ (0.05 and 0.082 charge/MW, respectively) compared to Na^+^ and K^+^ (0.044 and 0.026), which impedes their penetration (Armenteros et al., 2009). The salt content in the K25Ca15 substitution group showed no significant difference from the control group (*p* > 0.05), whereas the salt content in the K25Mg15 substitution group was significantly lower than that that in the control group (*p* < 0.05). This indicates that the co-substitution of NaCl with KCl and MgCl_2_ can significantly reduce the final salt content in sour meat.

### 3.2 Proteolytic activity dynamics during sour meat fermentation

#### 3.2.1 Variations in cathepsin activity

**Fig. 2:**
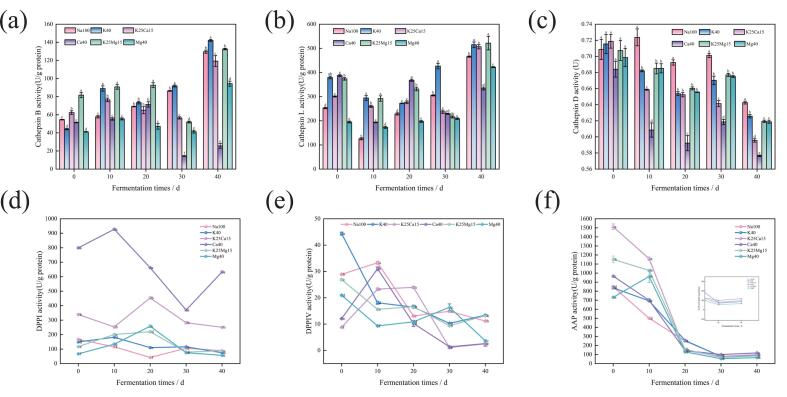
Changes of endogenous proteases activity of sour meat during fermentation. (a) Cathepsin B; (b) Cathepsin L; (c) Cathepsin D; (d) DPPI; (e) DPPIV; (f) AAP. Note: Different lowercase letters indicate significant differences at *p* < 0.05.

The activities of cathepsins during the fermentation of sour meat are shown in Fig. 2 Both cathepsin B and cathepsin L remained relatively active throughout the entire process, with the activity of cathepsin L being substantially higher than that of cathepsin B. This indicates that cathepsins made significant contributions to proteolysis, which aligns with previous reports (Zhang, Bi, et al., 2024). Furthermore, the activities of both cathepsin B and cathepsin L reached their highest levels at day 40, which differs from the conventional understanding that endogenous enzyme activities typically decline during the later stages of fermentation due to protein degradation and oxidative inactivation. This phenomenon may be attributed to the gradual disruption of cellular structures during fermentation, which releases a large amount of cathepsins originally confined within lysosomes into the sarcoplasm. This release increases the total amount of extractable enzyme protein, thereby resulting in higher measured activity in the in vitro assay (G.R. O'Halloran, 1997). On days 0, 10, and 20, cathepsin B activity in the K25Mg15 group exhibited significantly elevated levels compared to the control group (*p* < 0.05). Specifically, the cathepsin B activities in the K25Mg15 group were 149.52%, 156.72%, and 134.14% of those in the Na100 group at the respective time points. The addition of MgCl_2_ in fermented meat enhances the activity of cathepsin B, which is consistent with the finding of previous the previous study (Zhang, Yang, et al., 2020). These results demonstrate that partially replacing NaCl with KCl and MgCl_2_ can significantly enhance cathepsin B activity in sour meat. It is noteworthy that the cathepsin B activity in the K40 substitution group was significantly higher than in the Na100 group on days 10, 20, 30, and 40 (*p* < 0.05). Conversely, the activity in the Ca40 substitution group was significantly lower than in other experimental groups on days 30 and 40. This suggests that a high concentration of K^+^ ions can enhance cathepsin B activity, whereas a high concentration of Ca^2+^ ions strongly inhibits it.

As observed in Fig. 2b, on day 40, the cathepsin L activities in the K40, K25Ca15, and K25Mg15 substitution groups were all significantly higher than in the Na100 group (*p* < 0.05). The cathepsin L activities in the K40, K25Ca15, and K25Mg15 groups were 110.56%, 108.81%, and 111.97% of the Na100 group activity at that time, respectively. Furthermore, the Ca40 group showed significantly lower activity than all other groups, indicating that a high concentration of calcium ions also significantly suppresses cathepsin L activity. In summary, the results indicate that a high concentration of K^+^ ions, as well as the partial replacement of NaCl using KCl combined with divalent salts (CaCl_2_ or MgCl_2_), can significantly increase cathepsin L activity in sour meat.

The changes in cathepsin D activity during the fermentation of sour meat are presented in Fig. 2c. The activity of cathepsin D exhibited a fluctuating but overall downward trend throughout the process. The cathepsin D activities in all salt-substitution groups were significantly lower than that in the control group (*p* < 0.05). Notably, the K25Ca15 and Ca40 substitution groups exhibited the lowest cathepsin D activity among all experimental groups. These results indicate that the partial replacement of NaCl with non‑sodium salts, such as KCl, CaCl_2_, and MgCl_2_, inhibits cathepsin D activity in sour meat. Furthermore, the Ca^2+^ ion appears to have the most pronounced suppressive effect on the enzyme's activity.

#### 3.2.2 Activity profiles of dipeptidyl peptidase and AAP during sour meat fermentation

Fig. 2d illustrates the variations in DPP I activity throughout the sour meat processing. DPP I activity remained elevated during the entire process. The activities in the K25Ca15 and Ca40 substitution groups were significantly higher than those in the other experimental groups (*p* < 0.05), indicating a pronounced enhancing effect of Ca^2+^ ions on DPP I activity. The changes in DPP IV activity during sour meat processing are presented in Fig. 2e. The DPP IV activities across all experimental groups exhibited a fluctuating downward trend. Throughout the experimental period, activity values ranged from 2.48 U/g protein to 44.36 U/g protein. It is noteworthy that DPP IV activity was consistently and significantly lower than DPP I activity across all treatment groups, a trend that aligns with prior findings in dry-cured ham study (Zhao, Zhou, Xu, et al., 2005). The relatively weak inhibitory effect of NaCl on DPP I activity may contribute to the sustained high activity of DPP I during sour meat fermentation. On day 40, the DPP IV activities in the K40 and K25Mg15 substitution groups were significantly higher than that in the Na100 group (*p* < 0.05), whereas the activities in the Mg40, K25Ca15, and Ca40 groups were significantly lower than the Na100 group (*p* < 0.05). These results suggest that partial replacement of NaCl with KCl and CaCl_2_ significantly enhances DPP I activity in sour meat, while partial substitution with KCl and MgCl_2_ can improve DPP IV activity.

AAP accounts for 80%–83% of the total aminopeptidase activity in muscle (Zhao, Zhou, Tian, et al., 2005). Fig. 2f shows the changes in AAP activity during sour meat fermentation. AAP activity decreased sharply between day 10 and day 20, then stabilized. This trend is likely associated with the pH drop below 5.0 by day 20, which falls outside the optimal pH range for AAP (pH 5.0–7.0). On day 40, the AAP activities in the K25Ca15 and Ca40 substitution groups were significantly higher than that in the Na100 group (*p* < 0.05). These findings indicate that partially replacing NaCl with KCl and CaCl_2_ significantly increases AAP activity in sour meat.

Based on the protease activity changes and the proteolytic cascade, the regulatory effects of different salt substitutions on flavor precursor generation can be further clarified. In the K25Mg15 group, enhanced activities of cathepsins and DPP IV suggest a regulatory role at the upstream and midstream stages, favoring polypeptide-to-small peptide conversion. The K25Ca15 group showed increased DPP I and AAP activities, indicating regulation at the midstream and downstream stages, thus promoting the conversion of small peptides into FAAs.

### 3.3 Changes in proteolysis during sour meat fermentation

#### 3.3.1 Changes in PI

**Fig. 3:**
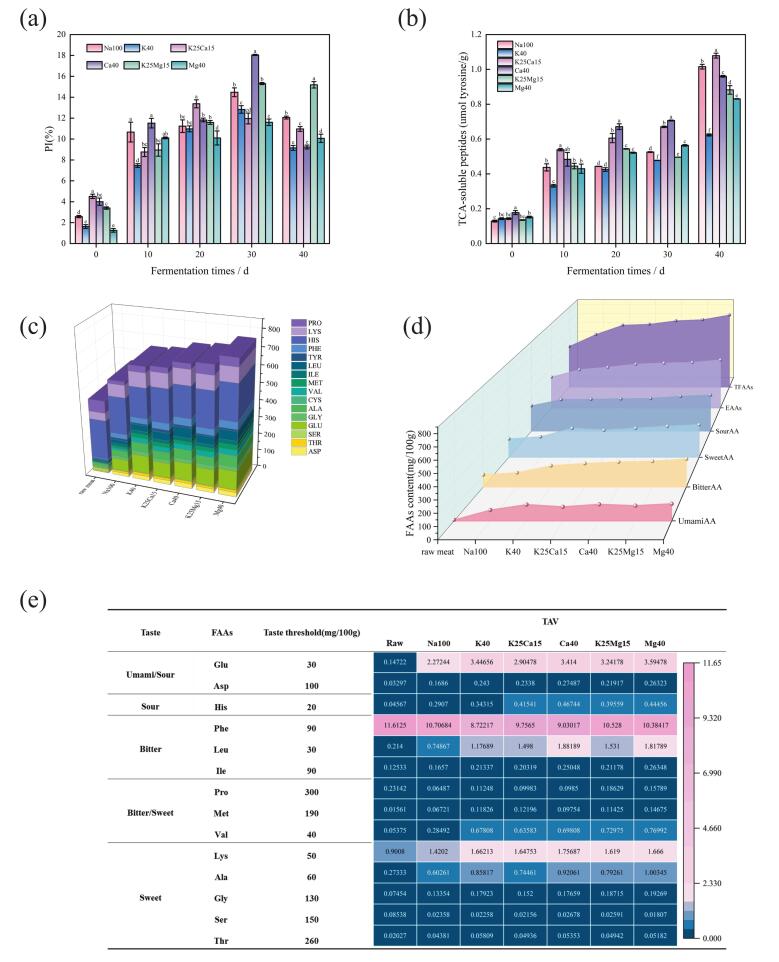
Proteolysis changes of sour meat. (a) Changes of PI; (b) Changes of TCA-soluble peptide content; (c) changes of FAAs; (d) Changes of taste-active amino acids; (e) TAV of amino acids EAAs is the sum of Ile, Met, Val, Phe, Leu, Trp, Thr and Lys. Sweet AA is the sum of Ala, Gly, Thr, Ser, Pro, Val, Met and Lys. Bitter AA is the sum of Leu, Val, Ile, Met, Phe, Tyr, Pro and Arg. Umami AA is the sum of Asp and Glu. Acid AA is the sum of Glu, Asp and His. Note: Different lowercase letters indicate significant differences at *p* < 0.05.

Proteases play a crucial role in the formation of characteristic flavors in fermented meats by hydrolyzing proteins into polypeptides and FAAs, which act as important flavor precursors (Toldrá, 2006). The PI is widely used as an indicator of proteolytic intensity (Harkouss et al., 2012). Fig. 3a illustrates the changes in the PI during the processing of sour meat. The PI displayed a trend of rising initially and then falling. This may be attributed to the low activities of cathepsin D, DPP IV, and AAP at day 40, which slowed down the rate of protein degradation (Petrova et al., 2016). Additionally, the decline in PI may also result from the consumption of free amino groups through their conversion into volatile flavor compounds (e.g., via the Maillard reaction and Strecker degradation). Thus, the late-stage PI decrease likely reflects both reduced proteolysis and increased amino group utilization for flavor formation. At day 0, the PI was significantly higher in the K25Ca15 and Ca40 groups (*p* < 0.05), when compared with the other experimental groups. The reason for this phenomenon may be that the K25Ca15 and Ca40 substitution groups significantly enhance the activities of DPP I and AAP, both of which exhibit relatively high initial activity levels at day 0. By day 40, the PI of the K25Mg15 group was significantly higher than that of the other groups (*p* < 0.05). This is likely due to the synergistic substitution of NaCl with KCl and MgCl_2_, which significantly enhanced the activities of cathepsins B and L enzymes that reached their peak activity precisely at 40 days of fermentation. Therefore, the PI in sour meat results from the combined action of multiple endogenous proteases, and its trend aligns well with the observed variations in their activities (Fig. 2). These findings further confirm that partially replacing NaCl in sour meat with KCl in combination with divalent salts (CaCl_2_ or MgCl_2_) can promote protein degradation.

It should be noted that during sour meat fermentation, lactic acid bacteria and other microorganisms may also secrete extracellular proteases and peptidases that participate in protein degradation. This study only measured the activities of endogenous proteases and did not distinguish or quantify the contribution of microbial-derived enzymes. Although previous studies have demonstrated that endogenous proteases play a dominant role in the proteolysis of fermented meat products (Verplaetse, 1994), the present study did not employ experimental approaches such as sterile fermentation or microbial inhibitors to exclude the interference of microbial enzymes. Therefore, the proteolytic effects observed in this study may reflect the combined action of both endogenous and microbial enzymes.

#### 3.3.2 Changes in TCA-soluble peptides during sour meat fermentation

The level of TCA-soluble peptides reflects the concentration of small peptides and correlates with the extent of proteolysis (Zhang, Han, et al., 2024). Fig. 3b illustrates the variation in TCA-soluble peptides during the fermentation of sour meat. The content of TCA-soluble peptides gradually increased with prolonged fermentation time. The K25Ca15 group exhibited significantly elevated TCA-solubles peptides levels compared to the Na100 group at days 10, 20, 30, and 40 (*p* < 0.05), reaching 123.23%, 136.57%, 127.57%, and 106.22% of the values in the Na100 group at the corresponding time points, respectively. This result confirms that partial replacement of NaCl with KCl and CaCl_2_ effectively promotes the accumulation of TCA-soluble peptides, further supporting the role of this specific salt substitution in enhancing protein degradation in sour meat.

#### 3.3.3 Changes in amino acids in sour meat

Based on preliminary observations of protein degradation through the PI and TCA-soluble peptides content, the process was further evaluated by analyzing amino acid content. Amino acids serve as key flavor constituents and precursors. They primarily generate volatile compounds via Strecker and Maillard pathways, significantly shaping the sensory profile of fermented meats (Wang, Dong, et al., 2022). Fig. 3c illustrates the changes in total free amino acids (TFAAs) content between raw meat and sour meat fermented for 40 days. The TFAAs content in raw meat was 431.51 mg/100 g. After 40 days of fermentation, the TFAAs content was significantly higher than that in raw meat, with values of 558.83, 657.76, 667.24, 704.28, 715.75, and 765.60 mg/100 g across the different groups. The sharp rise in FAAs levels is largely attributable to peptide cleavage by proteases, marking the terminal phase of proteolytic degradation (Li et al., 2022).

Compared to the Na100 group, the TFAAs content in the K40, K25Ca15, Ca40, K25Mg15, and Mg40 substitution groups increased by 17.70%, 19.40%, 26.02%, 28.08%, and 37.00%, respectively. These findings suggest that substitution with non‑sodium metal salts appears to promote the accumulation of TFAAs, with the extent of the effect depending on both the substitution ratio and the specific cation species. The TFAAs content increased with a higher substitution ratio of divalent salts (CaCl_2_ or MgCl_2_). The observed differences may not only arise from the differential activation of peptidase activities by the two ions, but may also involve differential modulation of substrate recognition selectivity by divalent cations. Many aminopeptidases (e.g., AAP) belong to the metalloprotease family, with their active sites containing one or two metal ions that play crucial roles in catalysis. Substitution of the native metal ions in the active center with different metal ions can alter the charge distribution, spatial configuration, and coordination environment of the active site, thereby affecting the enzyme's recognition selectivity toward N-terminal residues of substrates. For example, Izidoro et al. (2010) have found that magnesium ions can significantly alter the conformation of Furin, thereby bidirectionally regulating its activity and substrate selectivity with high specificity. Additionally, Toshimasa et al. (1990) have found that the active site of sea urchin sperm aminopeptidase can change its substrate preference depending on the Ca^2+^ concentration in the reaction medium, showing an activating effect on leucine substrates while exhibiting competitive inhibition toward arginine or lysine substrates in the presence of Ca^2+^. Based on these findings, it is inferred that Mg^2+^ and Ca^2+^ may differentially bind to the catalytic centers or regulatory sites of peptidases, altering the release efficiency of different peptide substrates and thereby leading to the observed differences in FAAs content between the two groups. Therefore, the presence of magnesium favors the generation of FAAs. These FAAs not only contribute directly to taste but also serve as precursors for the Maillard reaction to produce new volatile compounds, thereby enhancing flavor development in fermented products (Toldrá & Flores, 1998).

FAAs were demonstrated to contribute to the taste of fermented meat products, which impart sweet, bitter, umami, acid taste (Zhao et al., 2016). Fig. 3d shows the changes in taste-active amino acids in sour meat after 40 days of fermentation. Consistent with the trend in total amino acid content, the substitution with non‑sodium metal salts significantly elevated the levels of essential amino acids, sour-taste amino acids, sweet-taste amino acids, bitter-taste amino acids, and umami-taste amino acids. These amino acids act synergistically to enhance sourness, sweetness, bitterness, and umami in sour meat, thereby improving the complexity of the product's flavor profile. However, an excessively high substitution ratio of divalent salts (CaCl_2_ or MgCl_2_) in the Ca40 and Mg40 substitution groups also increased the content of bitter-taste amino acids, which may negatively affect the sensory quality of the sour meat. The results demonstrate that partially replacing NaCl with KCl and divalent salts (CaCl_2_ or MgCl_2_) effectively increases the content of both total FAAs and various taste-active amino acids, thereby enhancing the overall flavor and improving the sensory quality of sour meat.

To further assess the flavor contribution of FAAs in sour meat, taste activity values (TAVs) were calculated, as shown in Fig. 3e. An FAA is considered to contribute significantly to taste when its TAV is greater than 1. After fermentation, the sour meat contained more FAAs with TAVs >1, including Glu, Leu, and Lys. Notably, Phe had a TAV >1 and was present at high concentrations in both raw meat and all experimental groups. These results indicate that these FAAs significantly contribute to the taste profile of sour meat. Among them, Glu and Lys are umami/sour and sweet-taste amino acids, respectively, while Leu and Phe are bitter-taste amino acids. Similarly, Glu and Leu have been established as key contributors to the taste and richness of Jinhua ham, with Glu being the dominant amino acid (Zhou et al., 2021).

### 3.4 Changes in VOCs in sour meat

**Fig. 4:**
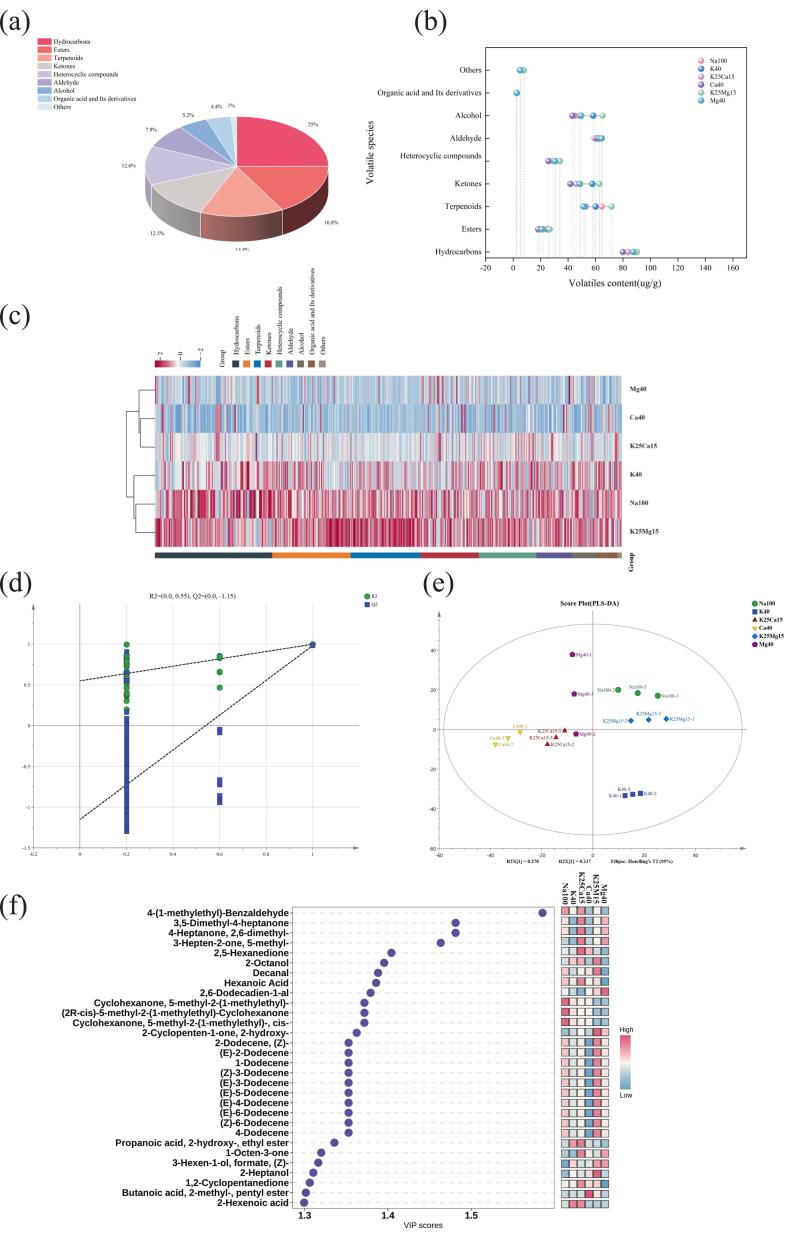
Changes of volatiles in sour meat. (a) Relative distribution of volatiles categories in sour meat; (b) Content of various volatiles in experimental groups; (c) Overall clustering diagram of samples (Red indicates high content, blue indicates low content); (d) Permutations diagram; (e) S-plots diagram; (f) VIP diagram. (For interpretation of the references to colour in this figure legend, the reader is referred to the web version of this article.)

To gain deeper insight into the impact of partially replacing NaCl with KCl and divalent salts on the flavor profile of sour meat, volatile compounds in the samples were systematically analyzed using HS-SPME/GC–MS. A total of 820 volatile compounds were qualitatively identified in sour meat samples fermented for 40 days. These compounds were categorized into hydrocarbons (205), esters (138), terpenes (121), ketones (103), heterocyclic compounds (101), aldehydes (65), alcohols (43), organic acids and their derivatives (36), and other categories (8). The relative content distribution of these VOCs is shown in Fig. 4a. Fig. 4b further illustrates the specific distribution of the relative contents of various VOCs across the experimental groups, demonstrating the modulating effect of different salt substitution strategies on the flavor compound profile. Cluster analysis (Fig. 4c) revealed that the overall composition pattern of VOCs in the K25Mg15 substitution group was similar to that of the Na100 group, and its total relative VOCs content was higher than that of the Na100 group. In contrast, the relative VOCs contents in the other substitution groups were generally lower than that in the Na100 group. These results indicate that the synergistic substitution of KCl and MgCl_2_ can maintain flavor characteristics similar to those of traditional sour meat, which may help preserve sensory acceptability, while also increasing the overall level of volatile flavor compounds, thereby enhancing sour meat flavor under reduced sodium content. This effect is closely associated with the synergistic enhancement of cathepsins B/L and DPP IV activities (Fig. 2). The coordinated increase in these enzymatic activities likely promotes the degradation of myofibrillar proteins, thereby releasing more amino acid precursors and providing abundant substrates for the formation of VOCs such as aldehydes, ketones, and esters.

To further investigate the impact of partial NaCl replacement with KCl and divalent salts on the volatile flavor profile of sour meat, a PLS-DA model was established. The model effectively revealed distinctions in VOCs among the six experimental groups and reflected the contribution of these compounds to sample differentiation. The model automatically fitted nine components, demonstrating high model fit indices (R^2^X = 0.972, R^2^Y = 0.997, Q^2^ = 0.952), indicating excellent model performance. A validation test with 200 random permutations was further conducted (Fig. 4b). The permutation results showed intercept values of R^2^ = 0.55 (< 0.6, green circles) and Q^2^ = −1.15 (< 0, blue squares), which were lower than the original corresponding points, confirming the model's robustness and absence of overfitting (Song et al., 2021).

As shown in Fig. 4e, the six sour meat groups were clearly separated in the PLS-DA score plot. The Na100, K40, and K25Mg15 groups clustered on the positive semi-axis, while the Mg40, K25Ca15, and Ca40 groups clustered on the negative semi-axis. These results indicate that both the type and proportion of non‑sodium metal salts used for NaCl replacement significantly influenced the volatile flavor profile of sour meat.

To identify key flavor-contributing compounds, variable importance in projection (VIP) scores were evaluated. Volatile compounds with VIP values greater than 1 were considered significant contributors to the overall flavor profile. Fig. 4f displays the top 30 VOCs in sour meat with VIP scores greater than 1, along with their contents in each experimental group, including 4-isopropylbenzaldehyde, 2,6-dimethyl-4-heptanone, and 2-octanol, indicating the significant contribution of these compounds to the flavor of sour meat. It should be noted that 4-isopropylbenzaldehyde primarily originates from the added Sichuan pepper rather than from fermentation-related biochemical pathways. However, different salt substitution formulations may influence the extraction or release of this compound from the spice into the meat matrix, potentially affecting its final concentration in sour meat products.

Although aldehydes generally account for a relatively low proportion of the total VOCs in sour meat, they are considered key contributors to the characteristic flavor profile of fermented meat products due to their low sensory thresholds (Dong et al., 2024). In the present study, aldehydes such as 4-isopropylbenzaldehyde, decanal, and (E,Z)-2,6-dodecadien-1-al were identified as key flavor compounds (VIP > 1), imparting spicy, fruity, waxy, and fatty aroma to the product. The relative content of aldehydes varied only slightly among the different salt substitution groups (Fig. 4b), indicating that partial replacement of NaCl with non‑sodium metal salts had limited influence on aldehyde formation and composition.

Esters are commonly present in the aroma profiles of fermented meat products and are mainly formed through alcohol-acid condensation reactions, often catalyzed by acyltransferases or esterases (Wang, Li, et al., 2022). The total ester content was highest in the K25Mg15 substitution group. Notably, the contents of ethyl octanoate, which imparts intense tropical fruit notes such as pineapple and pear, and ethyl lactate, which contributes creamy, buttery, and milky aromas, were higher in the K25Ca15 substitution group than in the other experimental groups. This may be attributed to the higher activities of DPP I and AAP in this group (Fig. 2). These enzymes promote the generation of small peptides and FAAs (such as leucine and valine), which serve as key precursors for the formation of the relevant branched-chain fatty acid esters. These results demonstrate that partially replacing NaCl in sour meat with KCl combined with divalent salts (CaCl_2_ or MgCl_2_) is a feasible strategy for enhancing desirable flavor attributes in fermented meat products.

Ketones typically impart fruity and creamy aromas, making significant contributions to the overall flavor profile of sour meat (Huang et al., 2022). They are primarily derived from lipid autoxidation, microbial metabolism, and pathways such as the Maillard reaction and Strecker degradation. The relative abundance of ketones varied significantly across the six treatment groups, reaching a maximum of 62.73 μg/g in the K25Mg15 formulation. This may be attributed to the higher activities of cathepsins B/L and DPP IV in the K25Mg15 substitution group (Fig. 2). These enzymes hydrolyze muscle proteins to generate small peptides and FAAs, which serve as key precursors that further influence the formation of ketone flavor compounds through pathways such as microbial transformation, Maillard reaction, and Strecker degradation. According to the PLS-DA results, ketones such as 2,6-dimethyl-4-heptanone, 3,5-dimethyl-4-heptanone, and 1-octen-3-one exhibited VIP scores greater than 1. Moreover, the relative contents of 2,6-dimethyl-4-heptanone, 3,5-dimethyl-4-heptanone, and 1-octen-3-one in the K25Ca15 substitution group were significantly higher than those in the other experimental groups. Among these, 1-octen-3-one is particularly noteworthy in food flavor chemistry due to its extremely low odor threshold and intense mushroom, earthy, and fungal notes. This compound is derived from the oxidation of 1-octen-3-ol, an unsaturated alcohol commonly found in fermented meat products that exhibits mushroom and floral aromas (Olivares et al., 2013). These findings indicate that substituting NaCl partially with KCl alongside divalent salts (CaCl_2_ or MgCl_2_) markedly alters the profile of ketone-based flavor compounds in sour meat.

The formation of alcohols primarily results from the degradation of linoleic acid in muscle tissue via lipoxygenase and hydroperoxidase pathways. Most alcohols contribute pleasant aromatic notes, such as sweet, fresh, fruity, floral, and herbal qualities, thereby enriching the overall volatile flavor profile of meat products. In sour meat, alcohols with VIP values greater than 1 include 2-octanol, 2-heptanol, and 3-methyl-1-hexanol. Similar to the trend observed for ketones, the alcohol content in the K25Mg15 substitution group was significantly higher than in the other experimental groups. These findings demonstrate that partially replacing NaCl in sour meat with KCl and MgCl_2_ effectively enhances the accumulation of alcohols.

Acids also serve as major contributors to the flavor profile of sour meat. Compounds such as hexanoic acid, 2-hexenoic acid, and (E)-3-hexenoic acid were identified as key flavor substances (VIP > 1), imparting cheesy, grassy, and fatty notes to the product. The presence of these fatty acids is an inherent outcome of the fermentation process. However, the acid levels across the six sour meat sample groups showed no significant variation.

Owing to their characteristically high odor thresholds, hydrocarbons generally exert negligible influence on the overall flavor of fermented meat products (Bosse et al., 2017). They primarily originate from lipid oxidation and accumulation in animal muscle tissues. The terpenes identified in sour meat originate primarily from the added Sichuan pepper and ginger, contributing to its complex aroma profile with notes of spice, wood, herb, citrus, and mint.

### 3.5 Relationship between protease activity, FAAs, and key flavor compounds in sour meat

**Fig. 5:**
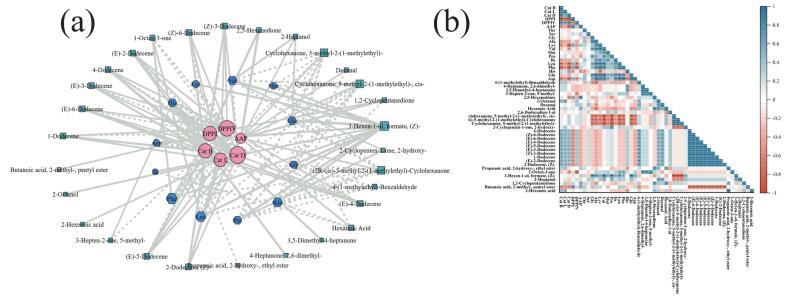
Correlation among endogenous proteases, key taste-active amino acids and key flavor substances in sour meat at day 40 of fermentation. (a) Network Diagram (Node size represents the connectivity of the compound, i.e., the number of substances with significant correlations to it. Higher connectivity is indicated by larger nodes. Solid lines represent positive correlations, while dashed lines represent negative correlations. Thicker lines correspond to larger absolute values of the correlation coefficient. Volatile compounds are shown in green, amino acids in blue, and proteases in pink.); (b) Correlation heatmap (* *p* < 0.05, ** *p* < 0.01, *** *p* < 0.001). (For interpretation of the references to colour in this figure legend, the reader is referred to the web version of this article.)

Endogenous protease activities play a crucial role in flavor formation during sour meat fermentation by modulating the extent of protein degradation, thereby influencing the final flavor profile and product quality. To elucidate these relationships, protease activities at day 40 of fermentation, 14 taste-active amino acids, and 30 major VOCs with high VIP values were selected for correlation analysis using Pearson correlation coefficients. Cluster heatmaps and correlation network diagrams were employed to visualize the interrelationships among these variables (Fig. 5).

The analysis revealed significant correlations between endogenous protease activities and various amino acids and flavor compounds, confirming their important role in shaping the flavor quality of sour meat. DPP I showed significant positive correlations (*p* < 0.05) with Asp, Phe, Leu, and Lys, suggesting a potential association with its potential role in enhancing sour, umami, bitter, and sweet taste characteristics. Cathepsin D was significantly positively correlated with His (*p* < 0.05). Conversely, the most pronounced negative correlations (*p* < 0.001) were observed between DPP IV and Phe, Cathepsin D and Asp, Phe and Lys, and Cathepsin B and Lys. Additional significant negative correlations (*p* < 0.05) included those between Cathepsin B and Ile, Lys, and Ala; Cathepsin L and Asp, Phe, and Leu; Cathepsin D and Val; DPP I and His; DPP IV and Asp and Lys; and AAP and His. These findings demonstrate that endogenous protease activities are significantly correlated with the amino acid composition in sour meat at day 40, consequently associating with its sourness, umami, sweetness, and bitterness.

The influence of endogenous protease activities on VOCs was also evident. Cathepsins B, L, and D showed significant positive correlations (*p* < 0.05) with C12 unsaturated alkenes including 4-dodecene, (Z)-6-dodecene, (E)-6-dodecene, (E)-4-dodecene, and (E)-5-dodecene. These structurally similar alkenes, generated through lipid oxidation during fermentation and maturation, collectively contribute faint fatty, waxy, and grass aromas to sour meat. In contrast, DPP I exhibited a significant negative correlation (*p* < 0.05) with these dodecene isomers. These results suggest that elevated cathepsin activity is correlated with promoted lipid oxidation in sour meat, thereby being associated with increased content of related volatile compounds. This may be attributed to the hydrolysis of muscle structural proteins and heme proteins by cathepsins. On the one hand, this disrupts the integrity of cell membranes, thereby increasing the contact area between lipid substrates (such as membrane phospholipids) and pro-oxidant factors. On the other hand, it releases and activates originally chelated metal ions (Fe^2+^) and heme, which act as potent catalysts, significantly promoting the initiation and propagation of lipid radical chain reactions. Notably, DPP I and AAP activities were negatively correlated with several VOCs. Given that the K25Ca15 and Ca40 substitution groups exhibited higher DPP I and AAP activities at day 40, this may help explain the relatively lower content of volatile compounds in these groups.

Additionally, 5-methyl-3-hepten-2-one showed a significantly negative correlation with DPP IV (*p* < 0.05). 2,5-Hexanedione exhibited significantly positive correlations with DPP I and AAP (*p* < 0.05), while showing a significantly negative correlation with cathepsin D (*p* < 0.05). 1-Octen-3-one demonstrated significantly negative correlations with cathepsin D and DPP IV (*p* < 0.05). 1,2-Cyclopentanedione was significantly positively correlated with AAP (*p* < 0.05). Previous research has indicated that Leu levels correlate with ketone content (Guo et al., 2021). Based on the results of this study, it is inferred that endogenous proteases in sour meat collectively regulate Leu accumulation, thereby being associated with the formation and accumulation of ketones. Cathepsins B and L showed significantly positive correlations with 2-octanol (*p* < 0.05), which imparts mushroom, earthy, slightly fatty, and herbal notes. Amyl 2-methylbutyrate was significantly negatively correlated with cathepsins (B, L, and D) (*p* < 0.05) but significantly positively correlated with DPP I and AAP (*p* < 0.05), contributing an intense and mature fruity aroma. 2-Hexenoic acid was significantly positively correlated with cathepsin L (*p* < 0.05), adding a raw, slightly green, and plant-like fatty nuance to the overall flavor matrix. These results demonstrate that endogenous protease activity is significantly correlated with the flavor profile of sour meat.

In summary, significant correlations exist between endogenous protease activities and specific amino acids and VOCs, indicating that the flavor profile of sour meat can be modulated through regulation of these protease activities.

## 4 Conclusion

This study systematically evaluated the effects of partially replacing NaCl in sour meat with the synergistic substitution of KCl and divalent salts (CaCl_2_ or MgCl_2_). The results demonstrate that co-substituting NaCl with KCl and CaCl_2_ or MgCl_2_ effectively modulates water distribution (increased Aw and reduced moisture content), promotes protein degradation, and elevates the levels of TFAAs and taste-active amino acids, thereby improving the flavor foundation of sour meat. Specifically, the KCl-MgCl_2_ combination markedly enhanced the activities of cathepsin B, cathepsin L, and DPP IV, effectively increasing the total content of VOCs. In contrast, the KCl-CaCl_2_ combination significantly elevated the activities of DPP I and AAP, promoting the accumulation of TCA-soluble peptides. Based on the PLS-DA model, key volatile compounds with VIP > 1, such as 4-isopropylbenzaldehyde, 2,6-dimethyl-4-heptanone, and 2-octanol, were identified as major contributors to the overall flavor profile. Correlation analysis further revealed significant associations between endogenous protease activities and certain amino acids and flavor compounds. In summary, the synergistic replacement of NaCl with KCl and divalent salts (CaCl_2_ or MgCl_2_) effectively regulates proteolytic and flavor formation pathways, thereby improving the comprehensive flavor quality of sour meat. This study provides a theoretical basis for developing sodium-reduced salt formulations and offers valuable insights for advancing traditional fermented meat products that combine health benefits with superior sensory attributes. Future studies should further examine the influence of different fermentation conditions to enhance the generalizability of the findings.

## CRediT authorship contribution statement

**Ying Chen:** Writing – original draft, Visualization, Formal analysis, Conceptualization. **Ning Liu:** Writing – review & editing, Visualization, Supervision, Formal analysis. **Yuqian Guan:** Writing – review & editing, Methodology, Formal analysis, Conceptualization. **Shenghui Bi:** Writing – review & editing, Data curation. **Lihua Shang:** Writing – review & editing, Investigation. **Chaoyue Yang:** Writing – review & editing, Conceptualization. **Cinan Li:** Software, Formal analysis, Data curation. **Yuanyuan Liu:** Validation, Methodology. **Binpeng Xu:** Visualization, Supervision. **Qiujin Zhu:** Visualization, Supervision, Methodology, Formal analysis. **Ying Zhou:** Writing – review & editing, Visualization, Supervision, Resources, Funding acquisition, Formal analysis, Conceptualization.

## Declaration of competing interest

The authors declare that they have no known competing financial interests or personal relationships that could have appeared to influence the work reported in this paper.

## Data Availability

Data will be made available on request.

## References

[bb0005] Aliño M., Grau R., Baigts D., Barat J.M. (2009). Influence of sodium replacement on the salting kinetics of pork loin. Journal of Food Engineering.

[bb0010] Armenteros M.n., Aristoy M.a.-C.n., Barat J.M., Toldrá F. (2009). Biochemical and sensory properties of dry-cured loins as affected by partial replacement of sodium by potassium, calcium, and magnesium. Journal of Agricultural and Food Chemistry.

[bb0015] Aspholm E., Lidman J., Burmann B. (2024). Structural basis of substrate recognition and allosteric activation of the proapoptotic mitochondrial HtrA2 protease. Nature Communications.

[bb0020] Bosse R., Wirth M., Konstanz A., Becker T., Weiss J., Gibis M. (2017). Determination of volatile marker compounds in raw ham using headspace-trap gas chromatography. Food Chemistry.

[bb0025] Brian Penner, S., Campbell, N. R. C., Chockalingam, A., Zarnke, K., & Van Vliet, B. (2007). Dietary sodium and cardiovascular outcomes: A rational approach. Canadian Journal of Cardiology*,* 23(7), 567–572. Doi: 10.1016/s0828-282x(07)70802-4. PMC2650761 17534464

[bb0030] Cittadini A., Domínguez R., Gómez B., Pateiro M., Pérez-Santaescolástica C., López-Fernández O., Lorenzo J.M. (2019). Effect of NaCl replacement by other chloride salts on physicochemical parameters, proteolysis and lipolysis of dry-cured foal “cecina”. Journal of Food Science and Technology.

[bb0035] Dong K., Wang Q., Li X., Li X., An F., Luo Z., Huang Q. (2024). An effective means to improve the flavor quality of traditional fermented sour meat: The salt reduction strategy. LWT-Food Science and Technology.

[bb0040] Guo X., Wang Y., Lu S., Wang J., Fu H., Gu B., Wang Q. (2021). Changes in proteolysis, protein oxidation, flavor, color and texture of dry-cured mutton ham during storage. LWT-Food Science and Technology.

[bb0045] Harkouss R., Mirade P.-S., Gatellier P. (2012). Development of a rapid, specific and efficient procedure for the determination of proteolytic activity in dry-cured ham: Definition of a new proteolysis index. Meat Science.

[bb0050] He F., MacGregor G. (2018). Role of salt intake in prevention of cardiovascular disease: Controversies and challenges. Nature Reviews Cardiology.

[bb0055] Hu J., La Vecchia C., Morrison H., Negri E., Mery L., Epidemiol C.C.R. (2011). Salt, processed meat and the risk of cancer. European Journal of Cancer Prevention.

[bb0060] Hu S., Xu X., Zhang W., Li C., Zhou G. (2023). Quality control of Jinhua ham from the influence between proteases activities and processing parameters: A review. Foods.

[bb0065] Huang Q., Dong K., Wang Q., Huang X., Wang G., An F., Luo P. (2022). Changes in volatile flavor of yak meat during oxidation based on multi-omics. Food Chemistry.

[bb0070] Izidoro M., Assis D., Oliveira V., Santos J., Juliano M., Lindberg I., Juliano L. (2010). Effects of magnesium ions on recombinant human furin: Selective activation of hydrolytic activity upon substrates derived from virus envelope glycoprotein. Biological Chemistry.

[bb0075] Li D., Liang Y., Xia Q., Pan D., Du L., He J., Zhou C. (2025). LC-MS/MS-based metabolomics and multivariate statistical analysis reveal the mechanism of yeast extracellular proteases on myofibrillar protein degradation, metabolite development and sensory characteristics improvement. Food Microbiology.

[bb0080] Li H., Wu J., Wan J., Zhou Y., Zhu Q. (2022). Extraction and identification of bioactive peptides from Panxian dry-cured ham with multifunctional activities. LWT-Food Science and Technology.

[bb0085] Li M., Zhu Q., Qu C., Gong X., Zhang Y., Zhang X., Wang S. (2024). Elucidation of potential relationship between endogenous proteases and key flavor substances in dry-cured pork coppa. Food Science and Human Wellness.

[bb0090] Liu Y., Li H., Li M., Liu L., Lu K., Bi S., Zhu Q. (2025). Study on protein hydrolysis and microbial community changes during the fermentation of pork loin ham mediated by electrical stimulation. Food Research International.

[bb0095] Lv J., Lin X., Liu M., Yan X., Liang H., Ji C., Zhu B. (2023). Effect of *Saccharomyces cerevisiae* LXPSC1 on microorganisms and metabolites of sour meat during the fermentation. Food Chemistry.

[bb0100] Meng D., Liu S., Zhao L., Li M., Zhu Y., Lee J.-H., Zhao G. (2025). Protein change based on spectral analysis: Effect of fermentation process on the degradation and multistage structure of sour meat proteins. Journal of Food Composition and Analysis.

[bb0105] G.R. O'Halloran, D. J. T., D.J. Buckley, W.J. Reville. (1997). The role of endogenous proteases in the tenderisation of fast glycolysing muscle. Meat Science*,* 47(3–4), 187–210. Doi: 10.1016/S0309-1740(97)00046-6. 22062733

[bb0110] Olivares A., Navarro J.L., Flores M. (2013). Characterization of volatile compounds responsible for the aroma in naturally fermented sausages by gas chromatography-olfactometry. Food Science and Technology International.

[bb0115] Petrova I., Tolstorebrov I., Mora L., Toldrá F., Eikevik T.M. (2016). Evolution of proteolytic and physico-chemical characteristics of Norwegian dry-cured ham during its processing. Meat Science.

[bb0120] Picon A., Nuñez M. (2022). Volatile compounds in high-pressure-treated dry-cured ham: A review. Meat Science.

[bb0125] dos Santos B.A., Campagnol P.C.B., Fagundes M.B., Wagner R., Pollonio M.A.R. (2015). Generation of volatile compounds in Brazilian low-sodium dry fermented sausages containing blends of NaC1, KC1, and CaC1 2 during processing and storage. Food Research International.

[bb0130] Song X., Canellas E., Nerin C. (2021). Screening of volatile decay markers of minced pork by headspace-solid phase microextraction–gas chromatography–mass spectrometry and chemometrics. Food Chemistry.

[bb0135] Sun Q., Chen Q., Li F., Zheng D., Kong B. (2016). Biogenic amine inhibition and quality protection of Harbin dry sausages by inoculation with *Staphylococcus xylosus* and *Lactobacillus plantarum*. Food Control.

[bb0140] Tian J., Han L., Yu Q., Shi X., Wang W. (2013). Changes in tenderness and cathepsins, activity during post mortem ageing of yak meat. Canadian Journal of Animal Science.

[bb0145] Toldrá F. (2006). The role of muscle enzymes in dry-cured meat products with different drying conditions. Trends in Food Science & Technology.

[bb0150] Toldrá F., Flores M. (1998). The role of muscle proteases and lipases in flavor development during the processing of dry-cured Ham. Critical Reviews in Food Science and Nutrition.

[bb0155] Toshimasa Y., Yokosawa H., Ishii S.I. (1990). Purification and characterization of an aminopeptidase from sperm of the sea urchin, *Strongylocentrotus intermedius*. Ca^2+^-dependent substrate specificity as a novel feature of the enzyme. The Journal of Biochemistry.

[bb0160] Verplaetse A. (1994).

[bb0165] Vidal V.A.S., Paglarini C.d.S., Ferreira A., Santos J.R.d., Pollonio M.A.R. (2020). Influence of the addition of KCl and CaCl_2_ blends on the physicochemical parameters of salted meat products throughout the processing steps. Food Science and Technology.

[bb0170] Wang H., Zhao S., Xia X., Liu J., Sun F., Kong B. (2024). Interaction of the extracellular protease from *Staphylococcus xylosus* with meat proteins elucidated via spectroscopic and molecular docking. Food Chemistry: X.

[bb0175] Wang Q., Dong K., Wu Y., An F., Luo Z., Huang Q., Wei S. (2022). Exploring the formation mechanism of off-flavor of irradiated yak meat based on metabolomics. Food Chemistry: X.

[bb0180] Wang Q., Li X., Xue B., Wu Y., Song H., Luo Z., Huang Q. (2022). Low-salt fermentation improves flavor and quality of sour meat: Microbiology and metabolomics. LWT-Food Science and Technology.

[bb0185] Wu H., Zhang Y., Long M., Tang J., Yu X., Wang J., Zhang J. (2014). Proteolysis and sensory properties of dry-cured bacon as affected by the partial substitution of sodium chloride with potassium chloride. Meat Science.

[bb0190] Zeng X., Meng J., Zhang W., He L., Deng L., Ye C. (2021). Changes in the microbiological, physicochemical properties of Chinese traditional fermented Suan rou at ripening fermentation. Food Science & Nutrition.

[bb0195] Zhang L., Han L., Yang J., Sun Q., Li K., Prakash S., Dong X. (2024). Preservation strategies for processed grass carp products: Analyzing quality and microbial dynamics during chilled and ice temperature storage. Food Chemistry: X.

[bb0200] Zhang T., Wang Y., Zhu J., Chen C., Jiang T., Fang S., Zhou C. (2025). TMT-labelled quantitative proteomics reveals the mechanism of *Rhodotorula mucilaginosa* on proteolysis of dry-cured ham: Structural protein degradation, amino acid release and taste improvement. Food Chemistry.

[bb0205] Zhang X., Bi S., Li M., Yue X., Wan J., Zhou Y., Zhu Q. (2024). Study on activation strategy and effect of protease activity during the post-processing stage of dry-cured meat based on electrical stimulation. Food Control.

[bb0210] Zhang X., Yang J., Gao H., Zhao Y., Wang J., Wang S. (2020). Substituting sodium by various metal ions affects the cathepsins activity and proteolysis in dry-cured pork butts. Meat Science.

[bb0215] Zhang Y., Hu P., Xie Y., Yang P., Zheng S., Tian Y., Feng D. (2020). DNA damage protection and antioxidant activities of peptides isolated from sour meat co-fermented by *P. pentosaceus* SWU73571 and *L. curvatus* LAB26. CyTA - Journal of Food.

[bb0220] Zhao C.J., Schieber A., Gänzle M.G. (2016). Formation of taste-active amino acids, amino acid derivatives and peptides in food fermentations – A review. Food Research International.

[bb0225] Zhao G.M., Zhou G.H., Tian W., Xu X.L., Wang Y.L., Luo X. (2005). Changes of alanyl aminopeptidase activity and free amino acid contents in biceps femoris during processing of Jinhua ham. Meat Science.

[bb0230] Zhao G.M., Zhou G.H., Xu X.L., Peng Z.Q., Huan Y.J., Jing Z.M., Chen M.W. (2005). Studies on time-related changes of dipeptidyl peptidase during processing of Jinhua ham using response surface methodology. Meat Science.

[bb0235] Zhong X., Bi S., Lan L., Liu M., Xiang W., Wan J., Zhu Q. (2025). Regulation of endogenous enzyme activity and quality of dry-cured pork loin at ripening stage by electrical stimulation. LWT - Food Science and Technology.

[bb0240] Zhou C.-Y., Bai Y., Wang C., Li C.-B., Xu X.-L., Pan D.-D., Zhou G.-H. (2021). ^1^H NMR-based metabolomics and sensory evaluation characterize taste substances of Jinhua ham with traditional and modern processing procedures. Food Control.

